# Proteomic analysis of kidneys from selenoprotein M transgenic rats in response to increased bioability of selenium

**DOI:** 10.1186/1559-0275-10-10

**Published:** 2013-08-12

**Authors:** Jun Seo Goo, Yo Na Kim, Kyung Mi Choi, In Sik Hwang, Ji Eun Kim, Young Ju Lee, Moon Hwa Kwak, Sun Bo Shim, Seung Wan Jee, Chul Joo Lim, Je Kyung Seong, Dae Youn Hwang

**Affiliations:** 1 Department of Biomaterials Science, College of Natural Resources & Life Science, Life and Industry Convergence Research Institute, Pusan National University, Miryang 627-706, South Korea; 2 Laboratory of Developmental Biology and Genomics, College of Veterinary Medicine, BK21 Program for Veterinary Science, Seoul National University, Seoul 151-742, South Korea; 3 Department of Laboratory Animal Resources, National Institute of Food and Drug Safety, Korea FDA, Osong 363-700, Korea; 4 Interdisciplinary Program for Bioinformatics, Program or Cancer Biology and BIO-MAX Institute, Seoul National University, Seoul 151-742, South Korea

**Keywords:** Antioxidative protein, Kidney, Selenium, Selenoprotein M, Transgenic rat

## Abstract

**Background:**

To characterize changes in global protein expression in kidneys of transgenic rats overexpressing human selenoprotein M (SelM) in response to increased bioabivility of selenium (Sel), total proteins extracted from kidneys of 10-week-old CMV/hSelM Tg and wild-type rats were separated by 2-dimensional gel electrophoresis and measured for changes in expression.

**Results:**

Ten and three proteins showing high antioxidant enzymatic activity were up- and down-regulated, respectively, in SelM-overexpressing CMV/hSelM Tg rats compared to controls based on an arbitrary 2-fold difference. Up-regulated proteins included LAP3, BAIAP2L1, CRP2, CD73 antigen, PDGF D, KIAA143 homolog, PRPPS-AP2, ZFP313, HSP-60, and N-WASP, whereas down-regulated proteins included ALKDH3, rMCP-3, and STC-1. After Sel treatment, five of the up-regulated proteins were significantly increased in expression in wild-type rats, whereas there were no changes in CMV/hSelM Tg rats. Only two of the down-regulated proteins showed reduced expression in wild-type and Tg rats after Sel treatment.

**Conclusions:**

These results show the primary novel biological evidences that new functional protein groups and individual proteins in kidneys of Tg rats relate to Sel biology including the response to Sel treatment and SelM expression.

## Background

Sel is considered to be a ubiquitous trace compound in nature and has been proven to be essential for mammalian health [1]. Specifically, Sel plays a dietary antioxidant role in the human body despite its low content of about 14–20 mg, and it is now recognized as an essential component of the active sites of numerous selenoenzymes [2]. Sel has also been shown to have insulin-like effects both *in vitro* and *in vivo*. For example, incubation of rat adipocytes with Sel stimulates glucose transport activity due to the translocation of two types of glucose transporters, cAMP phosphodiesterase activity, and ribosomal S6 protein phosphorylation [3]. Furthermore, Sel has been shown to regulate the activities of various enzymes involved in glycolysis and gluconeogenesis in streptozotocin-induced diabetes rats, whereas regulation of these enzymes is not induced by insulin [4]. Ayaz *et al*. [5] reported that Sel treatment may prevent and alleviate the symptoms of diabetes in animal models exhibiting heart, kidney, and platelet defects. Further, Sel treatment has been reported to have insulin-like effects during glucose metabolism through stimulation of tyrosine kinase in the insulin-signaling pathway [6].

Several reports have shown that Sel has detoxification effects on various heavy metals in a variety of toxicological and biochemical processes [7]. Especially, Sel co-accumulates with mercury in tissues of various biotas and significantly decreases the toxicity of inorganic mercury when injected in its selenite form [8]. Further, Sel has a detoxifying and protective effect upon kidney function improving glomerular filtration rate and increasing creatinine clearance under various abnormal conditions including acute kidney injury and chronic kidney disease [9-11]. Therefore, the kidney can consider as important target organ to identify novel markers and regulation mechanism on the effect of protection and detoxification induced by Sel treatment. However, although the relationship between Sel and renal disease have received enormous interest from nephrologist, few studies have been conducted to investigate whether or not Sel treatment and SelM overexpression affect global protein expression in kidneys of Tg rats using 2-DE.

As demonstrated by our data, proteomic analysis using kidney extracts showed up-regulation of 10 proteins as well as down-regulation of three proteins in CMV/hSelM Tg rats following Sel treatment.

## Results

### Enhancement of antioxidant activity in kidney tissue from CMV/hSelM Tg rats

**Figure 1:**
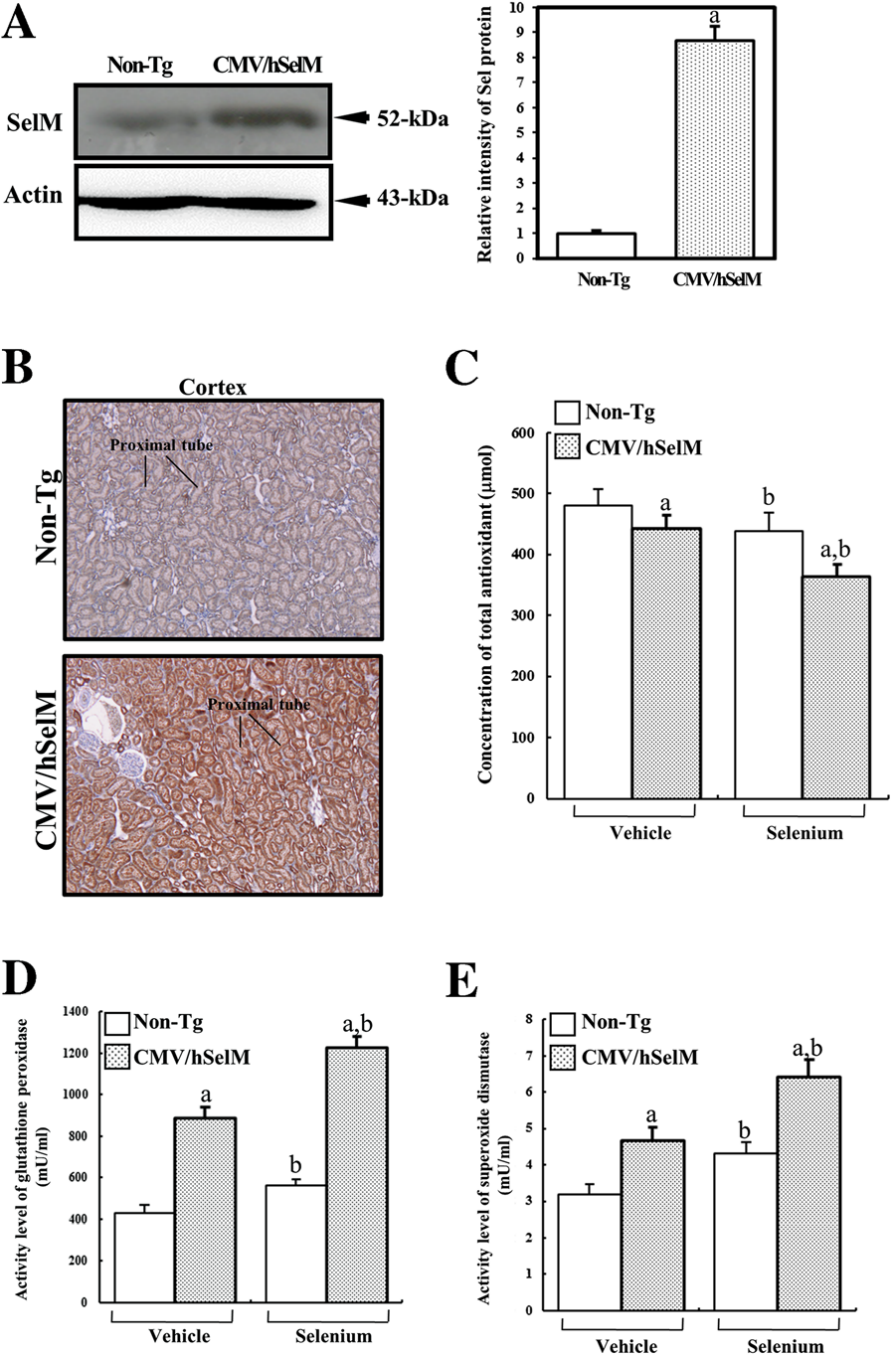
**Characterization of CMV/GFP-hSelM Tg rats. (A)** Expression of SelM proteins in kidney of CMV/hSelM Tg rats using antibody for both rat and human SelM. **(B)** Immunostaining analysis of SelM expression at 200x magnification. **(C)** Concentration of total antioxidants in serum by ELISA. Activities of GPx **(D)** and SOD **(E)** were detected in kidney tissues collected from CMV/EGFP-hSelM Tg and non-Tg rats. Six rats per group were assayed by ELISA. Data represent the mean **±** SD of three replicates. a, *p*<0.05 is the significance level compared with non-Tg rats. b, *p*<0.05 is the significance level compared with the vehicle-treated group.

To confirm whether or not alteration of antioxidative conditions is induced by Sel treatment and SelM overexpression in kidney tissue from Tg rats, SOD and GPx activities along with total antioxidants were measured in kidney tissues and sera. As shown in Figure 1A, a high level of hSelM protein was firstly detected by Western blotting using specific antibody in kidney tissue from CMV/hSelM Tg rats, whereas a low level of endogenous rat SelM protein was detected in non-Tg rats. Further, strong immunostaining intensity was observed throughout epithelial cells of proximal tubules in kidney tissue from CMV/hSelM Tg rats, although weak intensity was detected in the same region of non-Tg rats (Figure 1B).

Furthermore, the activities of SOD and GPx were higher in kidneys of CMV/hSelM Tg rats than those of non-Tg rats under vehicle treatment conditions. After Sel treatment, their levels further increased in both groups compared with those of vehicle-treated groups, although the rates of increase differed (Figure 1D and E). In addition, analysis of total antioxidants revealed the reverse pattern as that of the antioxidant enzymes SOD and GPx. Under vehicle treatment conditions, the antioxidant concentration was lower in serum of CMV/hSelM Tg rats compared to that of non-Tg rats. After Sel treatment, total antioxidants were significantly reduced in both CMV/hSelM Tg and non-Tg rats. However, they maintained their decrease ratio during Sel treatment (Figure. 1C). These results suggest that SelM overexpression and Sel treatment induced an increase in antioxidant protection in kidney tissues from CMV/hSelMTg rats.

### Effect of Sel treatment and SelM overexpression on global protein expression in kidney tissues

**Figure 2:**
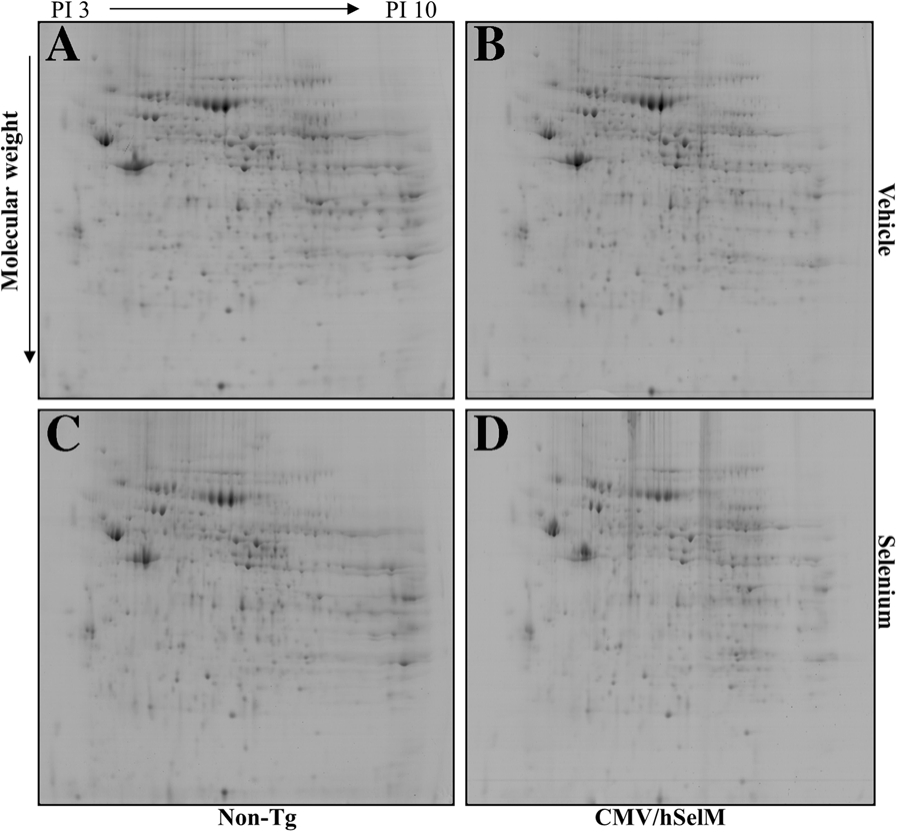
**2-DE protein patterns in kidney tissues from CMV/EGFP-hSelM Tg and non-Tg rats.** Kidney lysates (1 mg) from four groups, including vehicle-treated non-Tg rats **(A)**, Sel-treated non-Tg rats **(B)**, vehicle-treated CMV/hSelM Tg rats **(C)**, and Sel-treated CMV/hSelM Tg rats **(D)**, were subjected to one-dimensional IEF using 24- cm IPG strips in a pH range from 3–10 (nonlinear). Two-dimensional SDS-PAGE was performed on 8–18 % linear gradient acrylamide gels in an EttanDalt system. Protein spots were visualized by staining with Coomassie blue G-250.

To characterize changes in global protein expression in kidneys of CMV/hSelM Tg rats in response to the increased bioability of Sel and SelM, total proteins extracted from the cortex of 10-week-old CMV/hSelM Tg rats were separated by electrophoresis on a 2-DE gel and measured for changes in expression. Image analysis of the 2-DE gel showed good matching among the four analytical replicates, which included vehicle- and Sel-treated wild-type rats as well as vehicle- and Sel-treated Tg rats. In the 2-DE protein maps of the four group samples, approximately 300 spots were detected in one gel of kidney tissues (Figure 2). Of these, protein spots showing significant differences were selected for further analysis. Quantitative image analysis revealed 13 protein spots as key proteins that were differentially expressed among the four experimental groups; 10 spots were up-regulated while three spots were down-regulated in CMV/SelM Tg rats compared to wild-type rats of the vehicle-treated group. As shown in Table 1, the 10 up-regulated proteins were identified as LAP3, BAIAP2L1, CRP2, CD73 antigen, PDGF D, KIAA143 homolog, PRPPS-AP2, ZFP313, HSP-60, and N-WASP, whereas the three down-regulated proteins included ALKDH3, rMCP-3, and STC-1.

**Table 1:** List of differentially expressed proteins in the four experimental groups

**Spot No.**	**Protein name**	**Gene name**	**Accession No.**	**Sequence coverage (%)**	**Mw(Da)/pI**	**Mascot**	**Vehicle**		**Selenium**	
						**Score**	**non-Tg**	Tg*	non-Tg*	Tg*
1	Cytosol aminopeptidase (EC 3.4.11.1) (Leucine aminopeptidase) (LAP) (Leucyl aminopeptidase) (Leucine aminopeptidase 3) (Proline aminopeptidase) (EC 3.4.11.5) (Prolyl aminopeptidase)	Lap3	Q68FS4	42%	56115/6.77	25	1	2.3**±**0.24	1.8**±**0.31	1.3**±**0.09
2	Brain-specific angiogenesis inhibitor 1-associated protein 2-like protein 1 (BAI1-associated protein 2-like protein 1)(BAIAP2L1)	Baiap2l1	Q3KR97	46%	57432/8.95	29	1	2.1**±**0.17	1.7**±**0.38	1.5**±**0.12
3	Uncharacterized protein KIAA1143 homolog (KIAA1143 homolog)		Q5RKH3	52%	17442/8.51	28	1	2.4**±**0.13	1.5**±**0.26	1.3**±**0.2
4	5’-nucleotidase precursor (EC 3.1.3.5) (Ecto-5’-nucleotidase) (5’-NT) (CD73 antigen)	Nt5e	P21588	64%	63928/6.51	48	1	1.8**±**0.31	1.5**±**0.22	1.4**±**0.16
5	Cysteine and glycine-rich protein 2 (Cysteine-rich protein 2) (CRP2) Smooth muscle cell LIM protein) (SmLIM)	Csrp2	Q62908	64%	20926/8.95	27	1	1.9**±**0.16	1.6**±**0.15	1.5**±**0.24
6	Platelet-derived growth factor D precursor (PDGF D) (Iris-expressed growth factor) (Spinal cord-derived growth factor B) (SCDGF-B)	Pdgfd	Q9EQT1	35%	42782/8.11	25	1	2.3**±**0.35	1.6**±**0.21	0.9**±**0.13
7	Phosphoribosyl pyrophosphate synthetase-associated protein 2 (PRPP synthetase-associated protein 2)(PRPPS-AP2)(41 kDa phosphoribosypyrophosphate synthetase-associated protein) (PAP41)	Prpsap2	O08618	67%	40840/6.73	50	1	2.6**±**0.24	1.3**±**0.21	0.9**±**0.08
8	Zinc finger protein 313 (ZFP313)	Znf313	Q6J2U6	39%	25647/6.38	38	1	2.7**±**0.33	0.9**±**0.12	0.9**±**0.25
9	60 kDa heat shock protein, mitochondrial precursor (Heat shock protein 60) (HSP-60) (Hsp60) (60 kDa chaperonin) (Chaperonin 60) (CPN60) (Mitochondrial matrix protein P1) (HSP-65)	Hspd1	P63039	20%	60917/5.91	86	1	1.9**±**0.15	1.1**±**0.14	1.0**±**0.06
10	Neural Wiskott-Aldrich syndrome protein (N-WASP)	Wasl	O08816	27%	54291/8.33	33	1	3.2**±**0.34	1.2**±**0.21	0.9**±**0.25
11	Alpha-ketoglutarate-dependent dioxygenase alkB homolog 3 (EC 1.14.11.) (Alkylated DNA repair protein alkB homolog 3)(ALKDH3)	Alkbh3	Q5XIC8	60%	33990/8.53	30	1	0.5**±**0.06	0.3**±**0.04	0.4**±**0.03
12	Chymase precursor (EC 3.4.21.39) (Alpha-chymase) (Mast cell protease 3) (Mast cell protease III) (rMCP-III) (rMCP-3) (Mast cell protease 5) (rMCP-5)	Cma1	P50339	20%	27551/9.47	32	1	0.4**±**0.03	0.9**±**0.20	0.2**±**0.03
13	Stanniocalcin-1 precursor (STC-1)	Stc1	P97574	43%	27490/8.47	32	1	0.3**±**0.04	0.2**±**0.03	0.9**±**0.26

*The value in the last 3 columns on the right hand side was expressed as the relative value of spot volume for the non-Tg vehicle group which was defined as 1.

**Figure 3:**
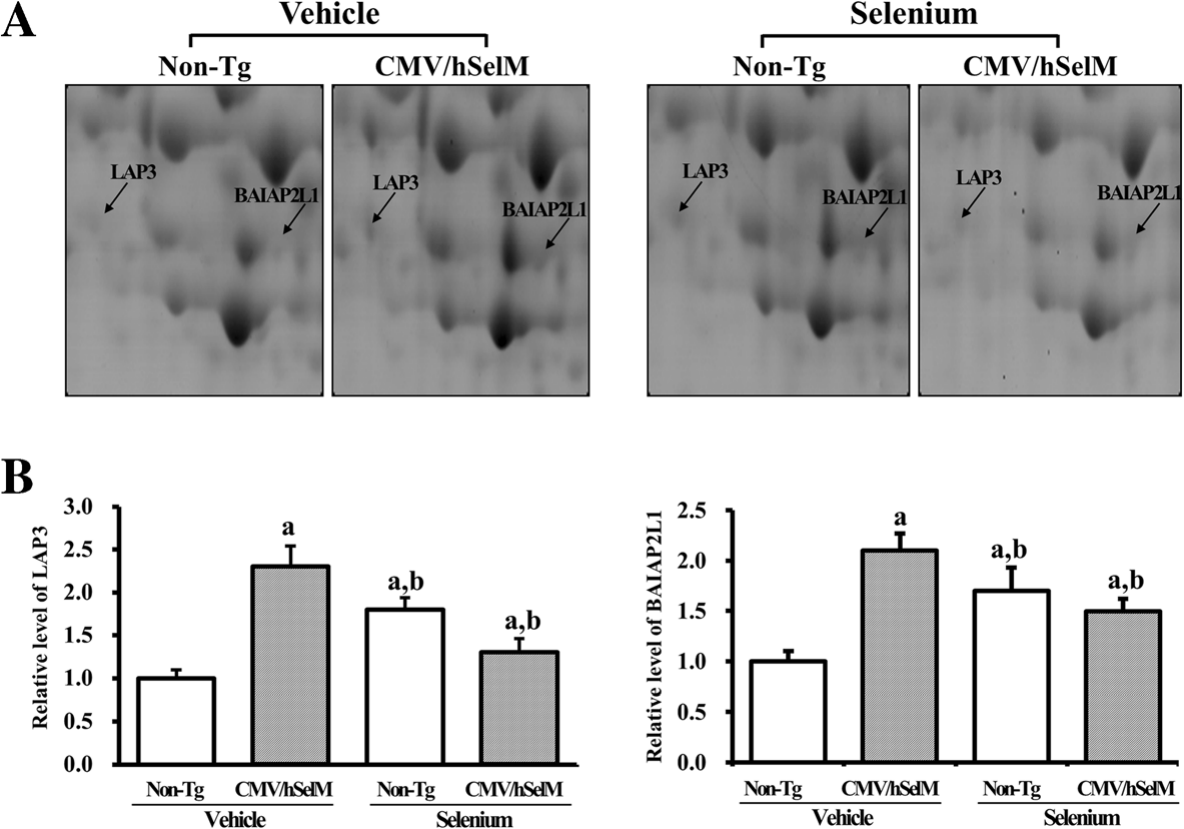
**Gel enlargement image showing LAP3 and BAIAP2L1 in kidney extracts. (A)** Up-regulated protein spots of LAP3 and BAIAP2L1 were detected in kidney extracts from the four experimental groups. Spots differentially expressed on 2-DE were further analyzed using a matrix-associated laser desorption/ionization time-of-flight (MALDI-TOF) mass spectrometer. **(B)** Expression levels of two proteins regulated by Sel treatment and SelM expression are represented relative to the non-Tg group. Data represent the mean **±** SD of three replicates. a, *p*<0.05 is the significance level compared with non-Tg rats. b, *p*<0.05 is the significance level compared with the vehicle-treated group.

**Figure 4:**
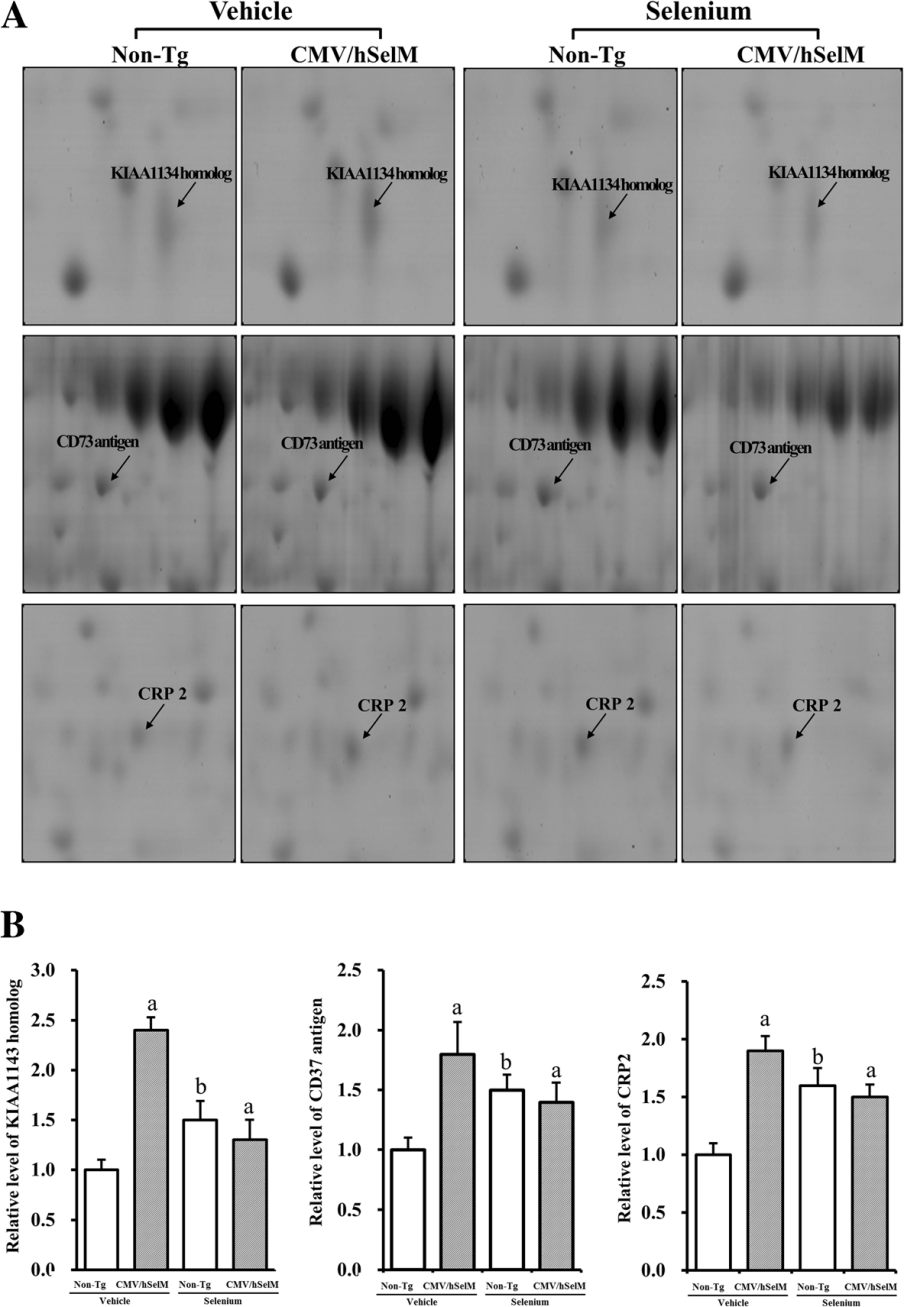
**Gel enlargement image showing KIAA1134 homolog, CD73 antigen, and CRP2 in kidney extracts. (A)** Up-regulated protein spots of KIAA1134 homolog, CD73 antigen, and CRP2 were detected in kidney extracts from the four experimental groups. Spots differentially expressed on 2-DE were further analyzed using a matrix-associated laser desorption/ionization time-of-flight (MALDI-TOF) mass spectrometer. **(B)** Expression levels of two proteins regulated by Sel treatment and SelM expression are represented relative to the non-Tg group. Data represent the mean **±** SD of three replicates. a, *p*<0.05 is the significance level compared with non-Tg rats. b, *p*<0.05 is the significance level compared with the vehicle-treated group.

After Sel treatment, these 13 protein spots showed differential expression patterns. According to their expression patterns, the 10 up-regulated spots were classified into four groups. Protein spots in the first group showed markedly increased expression upon Sel treatment and were identified as LAP3 and BAIAP2L1 (Figure 3). Under vehicle treatment conditions, the volume ratios of these two spots were significantly higher in CMV/hSelM Tg rats than in wild-type rats. After Sel treatment, their volumes were markedly increased by 100% in wild-type rats (Table 1), whereas CMV/hSelM Tg rats showed lower volume levels. The three spots in the second group exhibited medium changes in expression and were identified as uncharacterized protein KIAA143 homolog, CD73 antigen, and CRP2 (Figure 4). In the vehicle-treated group, the volumes of these spots were higher in CMV/hSelM Tg rats than in wild-type rats. However, Sel treatment significantly reduced spot volumes in wild-type rats, whereas expression levels of these proteins remained higher in CMV/hSelM Tg rats.

**Figure 5:**
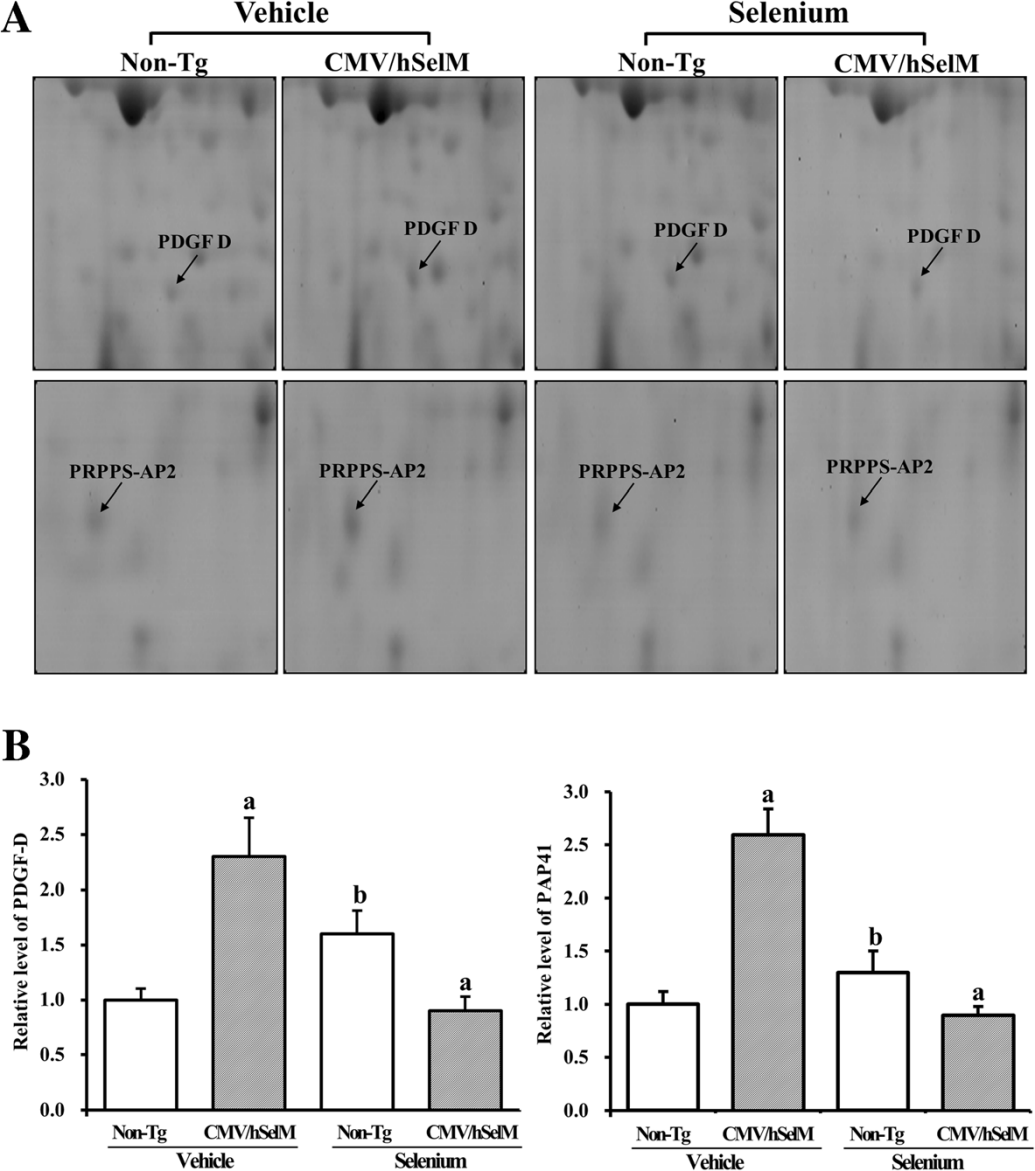
**Gel enlargement image showing PDGF D and PAP41 in kidney extracts. (A)** Up-regulated or maintained protein spots of PDGF D and PAP41 were detected in kidney extracts from the four experimental groups. Spots differentially expressed on 2-DE were further analyzed using a matrix-associated laser desorption/ionization time-of-flight (MALDI-TOF) mass spectrometer. **(B)** Expression levels of two proteins regulated by Sel treatment and SelM expression are represented relative to the non-Tg group. Data represent the mean **±** SD of three replicates. a, *p*<0.05 is the significance level compared with non-Tg rats. b, *p*<0.05 is the significance level compared with the vehicle-treated group.

**Figure 6:**
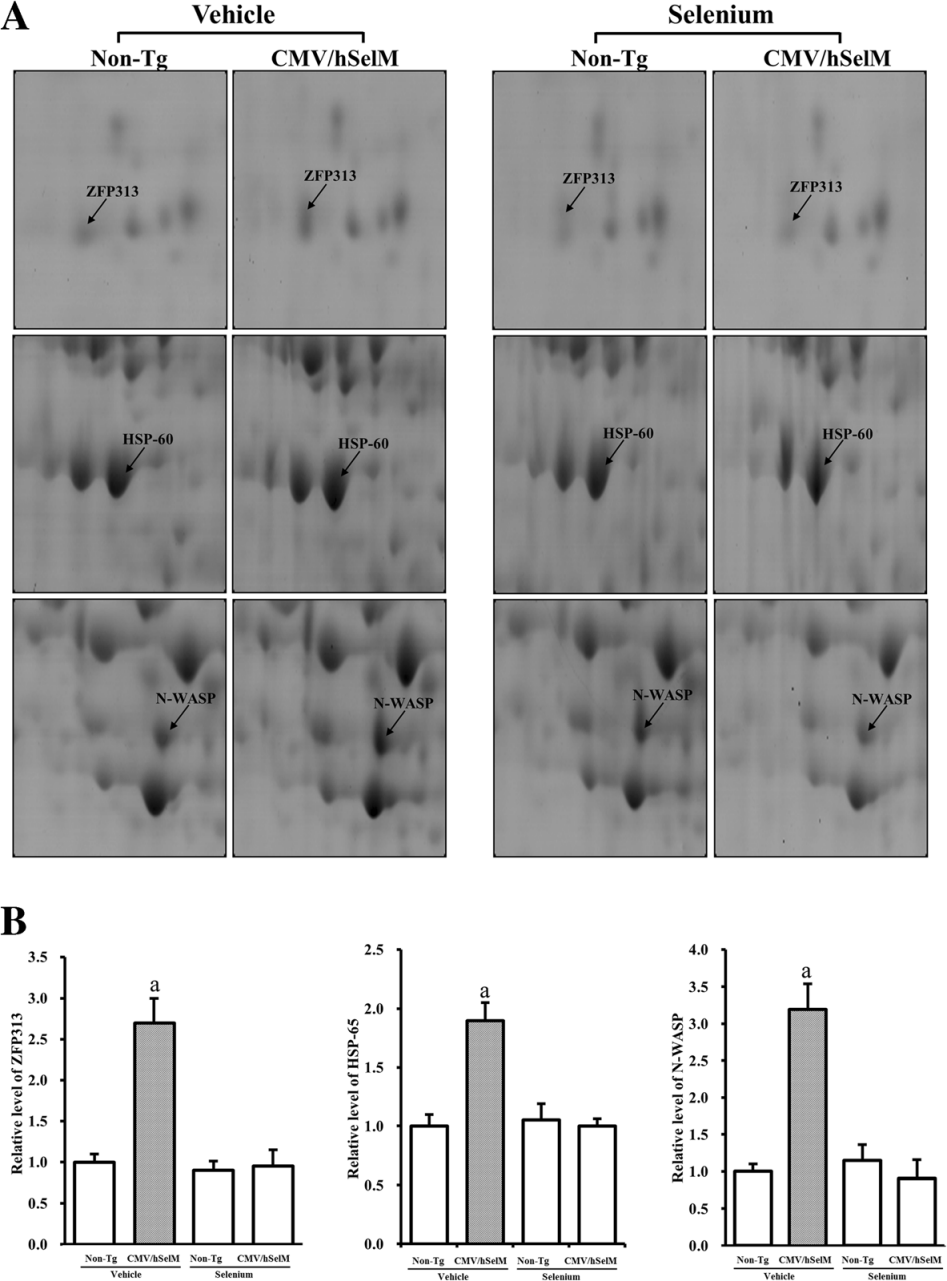
**Gel enlargement image showing ZFP313, HSP-60, and N-WASP in kidney extracts. (A)** Three spots (ZFP313, HSP-60, and N-WASP) up-regulated only by SelM overexpression were detected in kidney extracts from the four experimental groups. Spots differentially expressed on 2-DE were further analyzed using a matrix-associated laser desorption/ionization time-of-flight (MALDI-TOF) mass spectrometer. **(B)** Expression levels of two proteins regulated by Sel treatment and SelM expression are represented relative to the non-Tg group. Data represent the mean **±** SD of three replicates. a, *p*<0.05 is the significance level compared with non-Tg rats. b, *p*<0.05 is the significance level compared with the vehicle-treated group.

In the third group, two spots were identified as PDGF D and PAP41. Expression patterns of these spots were very similar with those of other up-regulated spots under vehicle treatment conditions (Figure 5). However, under Sel treatment conditions, the volumes of these spots were elevated only in wild-type rats, with spot volumes markedly reduced in CMV/hSelM Tg rats. Finally, in the fourth group, changes in protein expression were not affected by Sel treatment but instead induced only by SelM overexpression. This group included three spots identified as ZFP313, HSP-60, and N-WASP. These patterns were observed in both groups at the same time (Figure 6).

**Figure 7:**
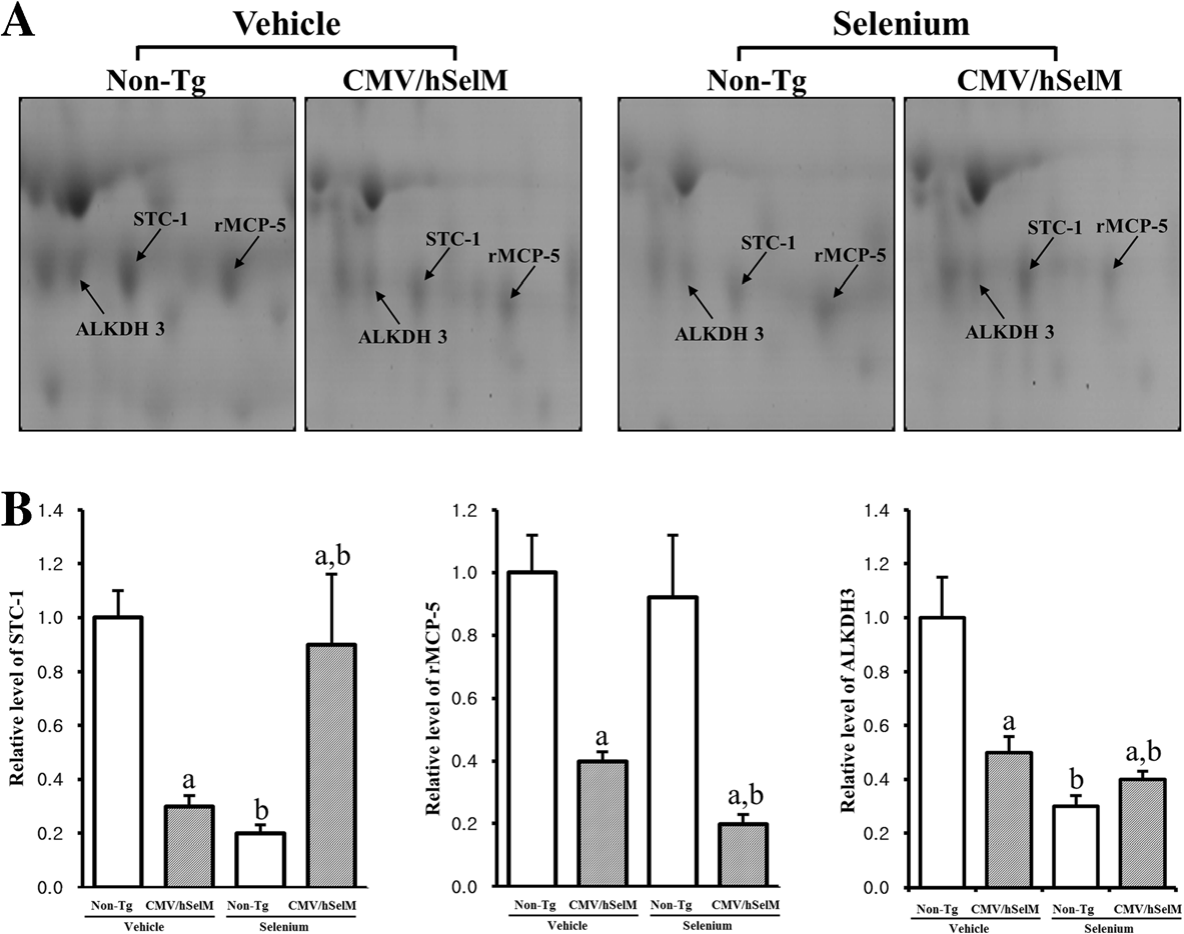
**Gel enlargement image showing STC-1, rMCP-5, and ALKDH3 in kidney extracts. (A)** Differentially regulated protein spots of STC-1, rMCP-5, and ALKDH3 were detected in kidney extracts from the four experimental groups. Spots differentially expressed on 2-DE were further analyzed using a matrix-associated laser desorption/ionization time-of-flight (MALDI-TOF) mass spectrometer. **(B)** Expression levels of two proteins regulated by Sel treatment and SelM expression are represented relative to the non-Tg group. Data represent the mean **±** SD of three replicates. a, *p*<0.05 is the significance level compared with non-Tg rats. b, *p*<0.05 is the significance level compared with the vehicle-treated group.

Treatment with Sel also induced differential expression patterns for down-regulated proteins. Firstly, a large spot was identified in 2-DE protein maps as ALKDH3. Under vehicle treatment conditions, ALKDH3 was expressed at a very low level in CMV/hSelM Tg rats compared to wild-type rats. On the other hand, Sel treatment induced down-regulation of ALKDH3 in wild-type rats, with a further decrease in CMV/hSelM Tg rats. A similar pattern was observed for another spot, which was identified as STC-1. The expression level of this protein was markedly decreased in wild-type rats after Sel treatment, whereas it did not change in CMV/hSelM Tg rats. Finally, Sel treatment induced a decrease in the spot volume of rMCP-5 in CMV/hSelM Tg rats, whereas wild-type rats showed no change (Figure 7).

Therefore, these results suggest that SelM overexpression and Sel treatment can induce changes in the expression levels of 13 major proteins that are related with blood volume and systemic vascular resistance, nucleotide metabolism, and kidney disease.

### Confirmation of AKLDH3, HSP60, and LAP3 expression

**Figure 8:**
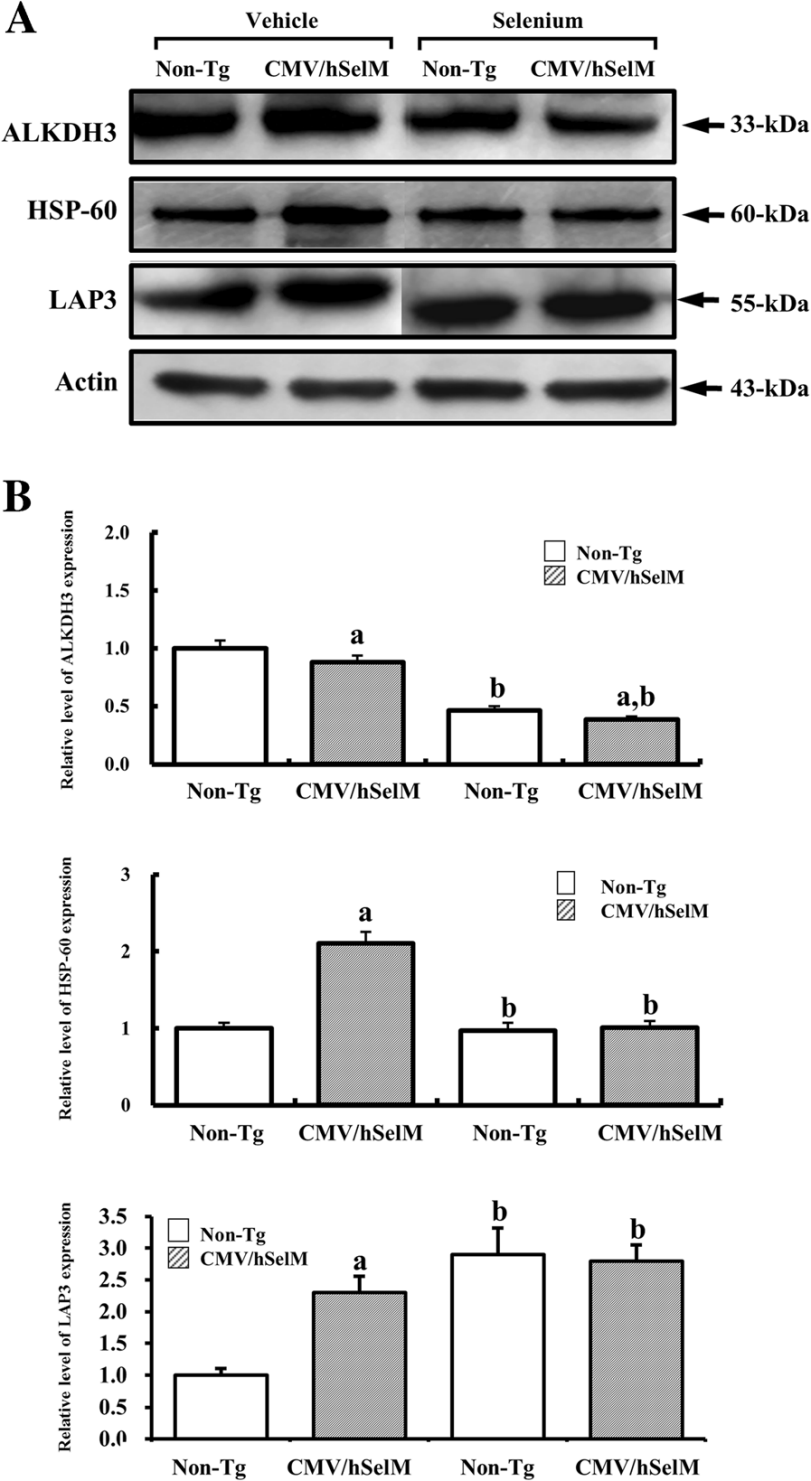
**Verification of ALKDH3, HSP-60, and LAP3 protein expression.** Nitrocellulose membranes transferring 50 μg of protein from kidneys of CMV/hSelM Tg and non-Tg rats were incubated with antibody specific for ALKDH3, HSP-60, LAP3, or actin, followed by horseradish peroxidase-conjugated goat anti-rabbit IgG. Data represent the mean **±** SD of three replicates. a, *p*<0.05 is the significance level compared with non-Tg rats. b, *p*<0.05 is the significance level compared with the vehicle-treated group.

Western blot analysis was performed to validate the changes in protein expression levels of three selected spots (AKLDH3, HSP-60, and LPA3**)** identified by 2-DE. To achieve this, we selected candidate genes according to the following criteria: 1) proteins showing a high magnitude fold change (LAP3), 2) proteins not annotated with function (AKLDH), and 3) proteins linked to various metabolic functions (HSP60). Accordingly, one up-regulated protein (LAP3), one maintained protein (HSP60), and one down-regulated protein (AKLDH3) were selected and analyzed. As shown in Figure 8, the expression level of AKLDH3 was significantly lower in CMV/hSelM Tg rats compared to non-Tg rats (*p*<0.041). After Sel treatment, its level further decreased in both groups (*p*<0.045), although its total expression pattern was maintained. In the case of HSP-60, its expression level was higher in CMV/hSelM Tg rats than in non-Tg rats under vehicle treatment conditions (*p*<0.008). However, expression of this protein significantly decreased in response to Sel treatment only in CMV/hSelM Tg rats (*p*<0.013), whereas non-Tg rats did not show any significant difference in expression. Furthermore, the LAP3 expression level was significantly increased in CMV/hSelM Tg and Sel-treated rats compared with non-Tg rats. The expression pattern of this protein in the Western blot analysis was very similar with that in the 2-DE gel image. These results suggest that the alteration of protein spots detected by 2-DE exactly reflects changes in protein expression in kidneys of both CMV/hSelM Tg and non-Tg rats.

## Discussion

SelM was recently reported as a new selenoprotein containing a 145-amino acid open reading frame along with an in-frame TGA as a Sec codon. Further, homologous SelM proteins have been found in various species, including rat, zebrafish, and other vertebrates, although Sec is conserved among these homologs [12]. Furthermore, SelM contributes to spicule formation in the demosponge *Suberitesdomuncula*[13]. In addition, this protein is tightly correlated with a suppressive or protective role in the pathology of patients with Alzheimer’s disease [14]. However, global changes in total protein expression have never been reported in kidney tissue from Tg rats overexpressing hSelM using 2-DE.

The human body produces several types Sel-dependent or antioxidant enzymes, including thioredoxin reductase, glutathione S-transferase, glutathione peroxidase, CuZn-superoxide dismutase, and Mn-superoxide dismutase [15]. Of these several enzymes, GPX is most abundant selenoprotein expressed in both the cytosol and mitochondria of mammalian cells, where it reduces H_2_O_2_ and organic hydroperoxides [16-18]. Further, Sel treatment was shown to induce increases in GSH-Px and SOD activities compared with control, although their increase ratios varied [19]. Our results in Figure 1 are in complete agreement with previous studies. Therefore, these results provide additional evidence that Sel treatment could induce antioxidant status in animals.

In this study, 2-DE analysis showed that SelM overexpression and Sel treatment were tightly associated with the expression of several proteins, including 10 up-regulated proteins and three down-regulated proteins. Of these proteins, LAP3 and BAIAP2L1 showed similar expression patterns after Sel treatment. Aminopeptidase is a homodimeric type II membrane-bound peptidase that specifically cleaves the N-terminal aspartyl residues from angiotensin II, resulting in angiotensin III [20]. It is also involved in the degradation of angiotensin I to form (des-Asp) Angiotensin I, which can be further cleavage to angiotensin III [21]. In the kidney, this enzyme is located in several regions, including the glomeruli, endothelial cells, mesangial cells, and tubular cells [22]. Furthermore, the activity of aminopeptidase increases in response to various divalent cations, particular calcium [21]. However, there are no reports on the correlation between aminopeptidase and Sel content. Our current results firstly show that aminopeptidase could be affected by SelM overexpression and Sel treatment.

The second group of three member proteins showed similar expression levels and included KIAA1143 homolog, CD73 antigen, and CRP2. Of these proteins, CD73 antigen is an immature form of 5’-nucleotidase (5’NT), which is a glycoprotein located primarily in the plasma membrane as an ectoenzyme and is composed of two identical subunits of 70–74 kDa [23]. Generally, 5’NT hydrolyzes extracellular nucleotides into membrane-permeable nucleosides [24]. Clinical analysis of the biochemistry of 5’NT has found that this enzyme is tightly associated with several diseases, such as hepatibiliary disease, primary tumors, hemolytic anemia, beta-thalassemia, lymphoma, and leukemia [25]. Especially, Imberti *et al*. showed that glutathione depletion is associated with a significant increase in serum 5’NT [26]. Moreover, 5’NT plays a critical role in tubuloglomerular feedback and rennin secretion in the kidney [27]. In this study, the expression level of CD73 antigen was differentially regulated by SelM overexpression and Sel treatment in both rat groups. Another protein identified as CRP2 is a cystein-rich protein (CRP) that interacts with cytoskeletal components such as α-actinin and zyxinin in vertebrates [28]. CRP2 is known as a novel protein that strongly suppresses fibroblast transformation induced by retroviral oncogenes or chemical carcinogens [29]. Recently, several studies have suggested that CRP is associated with alteration of cardiomyocyte thickness and hypertrophy [30]. However, until now, there has been no report on the function of this protein in the kidney. Our study results found that this protein could be induced by SelM overexpression and Sel treatment.

PDGF D and PRPPS-AP2 were classified into the third group. PDGF is comprised of four isoforms (A, B, C, and D) and two receptor chains (PDGFR-α and -β). Further, it is tightly associated with wound healing, atherosclerosis, fibrosis, and malignancy [31]. Of its four isoforms, PDGF B and D have been verified as key factors involved in mesangioproliferative disease and renal interstitial fibrosis [32]. Until now, many studies have suggested that PDGF is one of the most well characterized growth factors in renal disease [32]. Especially, the expression levels of PDGF and its receptor are significantly altered in renal disease, mesangial and interstitial proliferation, and in response to renal injury [32]. In our study, we found that SelM overexpression and Sel treatment could regulate the expression level of PDGF D in the rat kidney. In addition, PAP41 was detected as one spot showing a similar expression pattern under Sel treatment conditions. PRPP synthetase is composed of four different components, including two isoforms of 34-kDa catalytic subunits (PRS I and II) and two associated proteins of 39- and 41-kDa (PAP39 and PAP41). Of these components, rat PAP41 was first cloned from two expressed sequence tag (EST) clones, which are similar but not identical to PAP39, PRSI, or PRS II cDNA [33]. However, the functional mechanism and specific role of PAP41 in the kidney has not been elucidated, except for an association with X-linked dominant-inherited disorders and hyperuricemia [34]. Our 2-DE analysis results showed that the spot volume of PAP41 markedly increased upon SelM overexpression in kidneys of CMV/hSelM Tg rats, whereas Sel treatment may have affected PAP41 expression in kidneys of non-Tg rats.

The final group of up-regulated spots included three proteins, ZFP313, HSP-60, and N-WASP. The expression levels of these proteins were affected by SelM overexpression, whereas they remained unchanged upon Sel treatment. Of these proteins, HSP-60, a mammalian stress protein, is constitutively expressed and plays an essential function as a molecular chaperone under normal cellular conditions [35]. Especially, this protein is localized to the mitochondria and cytoplasm in rat kidneys at the electromicroscopic level [36]. Further, the expression level of this protein in the kidney can be altered by various factors such as high temperature, mercury chloride injection, transplantation, and osmolality [37]. However, there have been no reports on the effect of Sel on expression of HSP60. Finally, N-WASP, a member of the WASP family, regulates actin polymerization [38]. Moreover, this protein participates in normal brain development and synaptic plasticity [39]. In the kidney, N-WASP regulates hepatocyte growth factor-induced cell migration and invasion, which are required for epithelial tubulogenesis in the kidney [40]. Further, this protein significantly inhibits gentamicin accumulation in the mouse kidney. The above reports collectively suggest that N-WASP is associated with biological function of the kidney.

Other spots down-regulated by SelM overexpression were classified into another group that included ALKDH3, rMCP-3, and STC-1. Alkbh3 cDNA was firstly identified by the National Institutes of Health Mammalian Gene Collection (MGC) Program [41]. However, since then, there has been no report on the biological function of this gene. Therefore, our results provide information on the role of ALKDH3 in the kidney of rats. Finally, STC-1, a momodimer glycoprotein, is involved in calcium and phosphate regulation in mammals [42]. This protein is expressed in various tissues such as the pituitary gland, brain, kidney, liver, heart, muscle, and gonads. In our study, Sel treatment affected STC-1 expression in non-Tg rats, whereas there was no change in CMV/hSelM Tg rats.

## Conclusions

These results show a significant number of changes in proteins expression related to antioxidant protection in the kidney of CMV/hSelM Tg rats. Furthermore, several of our results are novel, as they have never been previously reported. Therefore, our results should increase our understanding of the detoxification mechanism as well as identify a novel target for protection of the kidney. However, intensive work is still needed to define the roles of SelM and Sel in protecting against antioxidant-related damage in the kidney.

## Methods

### Maintenance and identification of CMV/hSelM Tg rats

CMV/hSelM Tg rats used in this study showing high antioxidant status in the serum and erythrocytes were developed by microinjection of the CMV/hSelM fusion gene into fertilized rat eggs [43]. The animal protocol was reviewed and approved based on the ethical and scientific care procedures of the Korea Food & Drug Administration (Korea FDA)-Institutional Animal Care and Use Committee (KFDA-IACUC). All rats were kept in an accredited KFDA animal facility in accordance with AAALAC International Animal Care policies (Accredited Unit-Korea Food and Drug Administration: Unit Number-000996). The rats were given a standard irradiated chow diet (Purina Mills Inc.) *ad libitum* and maintained in a specified pathogen-free state (SPF) under a strict light cycle (lights on at 06:00 h and off at 18:00 h). All pedigrees were hemizygous for their transgene.

### Experimental design and Sel treatment

Sodium selenite (NaSeO_3_) purchased from Sigma (USA) was dissolved in distilled water to a final concentration of 0.2 μmol/μl [44,45]. Rats at 15 weeks of age were randomly divided into two subgroups (n=6) per group. The first subgroups of the CMV/hSelM Tg and non-Tg rat groups each received a comparable volume of distilled water daily via intraperitoneal injection (vehicle-treated CMV/hSelM Tg and non-Tg groups), whereas the second subgroups each received 5 μmol/kg body weight/day of sodium selenite via intraperitoneal injection for 3 weeks (Sel-treated CMV/hSelM Tg and non-Tg groups). Three weeks after Sel solution injection, the animals were immediately killed using CO_2_ gas, followed by extraction of blood from the abdominal vein and preparation of kidney samples.

### Western blot

Total proteins prepared from organs of CMV/hSelM Tg and non-Tg rats were separated by electrophoresis on a 4-20% SDS-PAGE gel for 3 h and then transferred to nitrocellulose membranes for 2 h at 40 V. Each membrane was incubated separately with anti-SelM antibody (Abcam), anti-ALKDH3 antibody (Santa Cruz Biotechnology Inc.), anti-HSP60 antibody (Cell Signaling Technology Inc.), anti-LAP3 antibody (Santa Cruz Biotechnology Inc.), or anti-actin antibody (Sigma) overnight at 4°C. The membranes were then incubated with horseradish peroxidase-conjugated goat anti-rabbit IgG (Zymed) at a 1:1,000 dilution at room temperature for 2 h. The membrane blots were developed using a Chemiluminescence Reagent Plus kit (ECL, Pharmacia).

### Analysis of GPx and SOD activities and total antioxidant concentration

The levels of GPx and SOD in kidneys of CMV/hSelM Tg and non-Tg rats were detected by following the calorimetric assay procedure using BIOXYTECH SOD-525 and BIOXYTECH GPx-340 kits (OxisResearch™, Portland, USA). The levels of total antioxidants in sera of CMV/hSelM Tg and non-Tg rats were detected by following the assay procedure using reagents in the Total Antioxidant Status Kit (Randox Labotatories Ltd., Antrim), as in the previous study [43].

### Sample preparation for 2-DE

Analyses of global protein expression by 2-DE were performed by following the methods established by our laboratory in previous studies [46]. Cortex samples isolated from kidney tissues were homogenized in liquid nitrogen, after which homogenized tissues were lysed in buffer (7 M urea, 2 M thiourea, 4% w/v CHAPS, 40 mM Tris, 100 mM DTE). Sample mixtures were centrifuged at 50,000 rpm at 4**°**C for 1 h. Protein concentrations were determined by the Bradford protein assay (Bio-Rad). In this process, cortex sample was made from a pool of each of the 6 animals in each group, and each pooled sample was run 3 times.

### 2-DE analysis

One-dimensional IEF was performed using 24-cm IPG strips (GE healthcare) in a pH range from 3–10 (nonlinear). Protein (1 mg) was loaded in a total volume of 450 μl, after which samples were diluted with rehydration solution (7 M urea, 2 M thiourea, 4 % w/v CHAPS, 40 mM Tirs, 100 mM DTE, 2 % IPG buffer 3–10). After rehydration for 13 h, the strips were focused at 30 V for 2 h, 100 V for 2 h, 200 V for 1 h, 500 V for 1 h, 1,000 V for 1 h, and finally at 8000 V for 22 h in order to obtain an approximately 100,000 VHr (IPGphor, GE healthcare). Once IEF was completed, the strips were equilibrated in 6 M urea containing 20 % glycerol, 2 % SDS, and 0.01 % bromophenol blue (BPB) with 10 mM tributyl phosphine (TBP). Two-dimensional SDS-PAGE was performed using 8–18 % linear gradient acrylamide gels on an EttanDalt system (GE healthcare). Proteins were visualized by staining with Coomassie blue G-250 (Bio-rad).

To analyze changes in protein expression between both types of mice according to SelM level, an average gel representing non-Tg mice was compared to an average gel representing CMV/hSelM Tg mice. Only those filtered spots exceeding an intensity threshold of a 1.5 or 2-fold increase or decrease between non-Tg and CMV/hSleM Tg mice were studied further, whereas the threshold regulation factor for the significance level was set at p≤0.05. Further, any spot showing a significant difference in expression between non-Tg and CMV/hSleM Tg mice was analyzed in all mice in order to map expression changes according to Sel-related factors. Furthermore, spots representing significant changes in expression were subsequently identified by mass spectrometry.

### Identification of protein spots

Stained gels were scanned on a GS800 densitometer (Bio-Rad) and analyzed using Image master™ (SIB, Sweden). The spots were digested using trypsin, after which supernatant peptide mixtures were loaded onto a Poros R2 column (Applied Biosystems) that had been washed with the following solutions: (1) 70 % acetonotrile in 5 % formic acid, (2) 100 % acetonitrile, and (3) 5 % formic acid. Peptides were eluted using 5 μl of α-cyano-4-hydroxycinnamic acid and analyzed by using a matrix-assisted laser desorption/ionization time-of-flight (MALDI-TOF) mass spectrometer (Voyager DE-PRO, Applied Biosystems). For protein identification, masses of peptides determined by MALDI-TOF were matched with theoretical peptides in the NCBI (http://www.ncbi.nih.gov/) database using the MASCOT (http://www.matrixscience.com) and ProFound programs (http://prowl.rockefeller.edu).

### Statistical analysis

Tests for significance between vehicle- and Sel-treated rats were performed using a One-Way ANOVA test of variance (SPSS for Windows, Release 10.10, Standard Version, Chicago, IL). Tests for significance between CMV/hSelM Tg and non-Tg rats were performed using a Post-Hoc test (SPSS for Windows, Release 10.10, Standard Version, Chicago, IL) of variance, and significance levels are given in the text. All values are reported as the mean ± standard deviation (SD). a *p* value < 0.05 was considered significant.

## Abbreviations

LAP3: Cytosol aminopeptidase; BAIAP2L1: Brain-specific angiogenesis inhibitor 1-associated protein 2-like protein; CD73 antigen: 5’-nucelotidase precursor; CRP2: Cysteine and glycine-rich protein 2; PDGF D: Platelet-derived growth factor D precursor; KIAA143 homolog: Uncharacterized protein KIAA143 homolog; PRPPS-AP2: Phosphoribosyl pyrophosphate synthetase-associated protein 2; ZFP313: Zinc finger protein 313; HSP-60: 60-kDa heat shock protein; N-WASP: Neural wiskott-aldrich syndrome protein; ALKDH3: Alpha-ketoglutarate-dependent dioxygenase, alkB homolog 3; rMCP-3: Chymase precursor; STC-1: Stanniocalcin-1 precursor.

## Competing interests

The authors declare that they have no competing interests.

## Authors’ contributions

JSG, YNK, KMC, ISH, JEK, YJL, MUK, JKS and DYH participated in the design of the study, animal experiment, 2D experiment and data analyses. SBS, SWJ and CJL helped the management and analysis of CMV/hSelM Tg rats. All authors read and approved the final manuscript.
